# XPO1, therapeutic … and prognostic target in sarcomas

**DOI:** 10.18632/oncoscience.304

**Published:** 2016-05-19

**Authors:** François Bertucci, Pascal Finetti, Daniel Birnbaum

**Affiliations:** Department of Medical Oncology, Institut Paoli-Calmettes, Marseille, France

**Keywords:** exportin 1, selinexor, soft tissue sarcoma, XPO1

In a recent issue of Oncotarget [1], Nakayama et al. showed the anti-tumor activity of selinexor, an inhibitor of exportin 1, against a wide variety of sarcoma preclinical models, including several pathological subtypes of soft tissue sarcomas (STS). STS, which amount to less than 2% of adult cancers are a heterogeneous disease with ∼50 different pathological subtypes [2]. Surgery is the main treatment of patients with early stage STS. Despite surgery, more than 40% of cases will experience metastatic relapse. The survival benefit of adjuvant anthracycline- based chemotherapy remains unproven today, perhaps in part because of the absence of accurate prognostic features and predictors of response to chemotherapy. Identifying new prognostic features, complementary and/ or more accurate than the current ones, is crucial. These may be molecular, such as the gene expression signature CINSARC [3]. In patients with metastasis not amenable to curative-intent surgery, the first-line treatment involves palliative chemotherapy, which has not changed over the three past decades and remains based on doxorubicin with or without ifosfamide. After intolerance or failure, the currently approved second-line therapies include chemotherapies (trabectedin, ifosfamide, dacarbazine) and a recently approved targeted therapy (pazopanib). Clearly, the improvement of systemic therapies is needed. The Nakayama's study represents a promising new avenue of research. Selinexor is a novel and safe oral small-molecule inhibitor of exportin 1 (XPO1/CRM1) with broad antitumor activity [4]. Exportin 1 is a karyopherin that mediates the nuclear export (and subsequent inactivation) of tumor suppressors such as P53, P73, BRCA, and P21. Exportin 1 is also involved in the activation of oncogenic pathways, through enhanced nuclear export of EIF4E, the sole transporter of guanine-capped mRNAs, including mRNAs for oncogenes such as MYC, cyclin D1, and MDM2. Thus, XPO1 inhibition provides a novel and promising anticancer strategy able to prevent inactivation of tumor suppressors and the translation of several key mRNAs and proteins by preventing their export and restricting them to the nucleus. XPO1 overexpression has been reported in different solid cancers and is associated with poor prognosis [5,6]. To our knowledge, XPO1 expression has never been assessed in clinical samples of STS.

**Figure 1:**
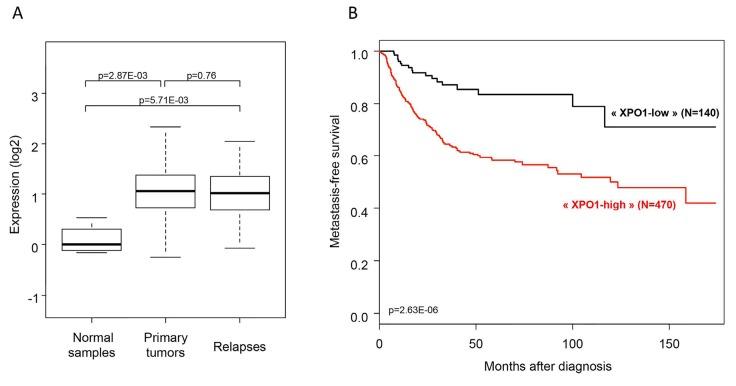
XPO1 expression in soft tissue sarcomas **A.** XPO1 mRNA expression level (log2) reported as a box plot according to the type of soft tissue samples (normal samples, STS primary tumors, STS relapses). The p-values are indicated (Mann Whitney test). **B.** Kaplan-Meier MFS curve in the 610 patients with STS, according to the XPO1 expression-based group (“XPO1-low” and “XPO1-high”; log-rank test).

We examined *XPO1* mRNA expression in a retrospective series of 1.398 clinical soft tissue samples gathered from 15 public data sets, including 1.362 primary tumors of STS, 32 relapses of STS, and 4 normal tissues. Expression profiles had been generated using DNA microarrays and RNASeq. Before analysis, expression data were normalized within and between all data sets as previously described [7]. XPO1 was significantly overexpressed in primary tumors when compared with normal samples (p=2.8E-03), and expressed at the same level in primary tumors and relapses (p=0.76; Figure 1A). We searched for correlations between XPO1 expression, assessed as binary and continuous variable, and clinicopathological features of the 1.362 non-metastatic and operated primary tumors. XPO1 expression was categorized in two groups (high versus low) according to the cut-off defined by ROC analysis of expression levels in primary tumors versus normal samples (AUC=0.93): 1.142 cases were included in the “XPO1-high” group and 220 in the “XPO1-low” group. When compared with the “XPO1-low” group, the “XPO1-high” group was associated with younger patients' age (p=3.0E-05, t-test), with pathological subtypes with more myxoid liposarcomas, leiomyosarcomas and other STS and less well differentiated/dedifferentiated and pleomorphic liposarcomas and undifferentiated sarcomas (p=8.0E-06, Fisher's exact test), and with high-risk CINSARC class (p=6.0E-07, Fisher's exact test). No correlation was found with patients' gender, pathological grade and tumor size, and depth location. Regarding prognosis, metastasis-free survival (MFS) data were available for 610 non-metastatic operated patients. The median follow-up was 36 months (range, 1-222) after diagnosis, 183 patients displayed a metastatic relapse, and the 5-year MFS was 64% (95%CI 59-69). XPO1 expression was associated with MFS with 58% 5-year MFS (95%CI 53-64) in the “XPO1-high” group versus 83% (95%CI 76-92) in the “XPO1-low” group (p=2.6E-06; Figure 1B), with respective median MFS of 38 months (range, 1-188) versus 33 months (range, 1-222). The hazard ratio for metastatic relapse was 2.97 (95%CI 1.84-4.79) in the “XPO1-high” group versus the “XPO1-low” group (p=7.5E-06, Wald test). In univariate analysis (Wald test), XPO1-based group (p=7.5E-06), pathological tumor type (p=4.30E-05), and CINSARC class (p=1.6E-11) were associated with MFS. In multivariate analysis, two variables, the CINSARC classification (p=8.4E-07) and the XPO1-based group (p=1.9E-03), remained associated with MFS, suggesting independent prognostic value. A similar prognostic analysis using *XPO1* expression as continuous variable showed the same result, with unfavorable independent prognostic value for high expression.

This analysis of *XPO1* expression in STS in such a large series of cancer samples nicely complements the study by Nakayama and colleagues and reinforces the potential therapeutic value of this new target in STS.
